# Development and validation of an experimental verbal Episodic Memory task in Spanish

**DOI:** 10.1590/2317-1782/20232022067en

**Published:** 2023-09-18

**Authors:** Gabriel Urrutia Urrutia, Pedro García Montenegro, Karina Carlesso Pagliarin, Márcia Keske-Soares

**Affiliations:** 1 Departamento de Ciencias de la Fonoaudiología, Facultad de Ciencias de la Salud, Universidad de Talca - UTALCA - Talca (VII Región del Maule), Chile.; 2 Programa de Pós-graduação em Distúrbios da Comunicação Humana, Universidade Federal de Santa Maria - UFSM - Santa Maria (RS), Brasil.

**Keywords:** Information Storage and Retrieval, Memory Consolidation, Episodic Memory, Semantics, Validity of Results, Cognitive Aging

## Abstract

**Purpose:**

To develop and validate an experimental verbal episodic memory task in Spanish.

**Methods:**

Six encoding blocks were elaborated, three deep and three superficial, each one with different demands of cognitive effort. The blocks were reviewed by four expert judges and tested in a pilot application. The agreement was assessed on whether the task allowed combined processing level and cognitive effort to be manipulated during incidental encoding of words, as well as clarity of instructions, examples, and workflow.

**Results:**

Variables such as lexical availability, metrics, and strength of association were useful to differentiate the cognitive effort between each block. The judges agreed that the processing blocks allowed a combined manipulation of the level of processing and cognitive effort and that the instructions are precise. After the pilot, the participants agreed that the instructions, examples, and way of working were easy to understand and perform.

**Conclusion:**

The results provide evidence of validity related to the content for the proposed experimental task, thus becoming a viable alternative to consider in research aimed at identifying environmental factors that contribute to compensating the defects shown by episodic memory with age.

## INTRODUCTION

Episodic memory (EM) is responsible for consciously encoding, storing, and retrieving past personally experienced events^(1)^. It is one of the first domains to show a decline since the onset of aging^(2)^, linked to inefficient functioning of the encoding and retrieval processes^(3,4)^.

Encoding is the memory process that participates in the acquisition and transformation of new information that is processed into a mental representation^(5)^. This process usually demands many processing resources such as the selective capacity of attention, processing speed, and executive control of working memory^(6)^. Unfortunately, with age, these processes tend to show a decline in their functioning^(7-9)^.

According to the limited resources hypothesis^(10)^, older adults (OA) would exhibit a lower capacity to spontaneously initiate relevant cognitive processing when encoding new information in memory.

According to the distributed cognition theory, cognitive performance would be the result of an interaction between internal (cognitive) and external (environmental) components^(11)^. According to such an approach, information processing would start “from the top down” mobilized by people's current intentions, but partly also by external stimulation that drives a “from the bottom-up” processing. Putting these ideas together, we can hypothesize that some age-related impairments in self-initiated (top-down) processing could be reduced by increasing the externally driven bottom-up component in the form of Environmental Support (ES)^(12)^.

Evidence from behavioral studies has shown that providing ES during encoding produces positive effects on recall^(13-15)^. The ES is understood as an external performance support that acts by manipulating the demands of a cognitive task to favor more efficient processing of information^(12)^.

In this line, two environmental factors have been proposed that could favor compensation mechanisms for EM, *cognitive effort,* and the *level of processing*.

The first refers to the proportion of processing that a person commits to a challenging task^(16)^. The level of processing refers to the degree of depth in which the information is processed, varying from a superficial level, which assists in more perceptual elements, to a deeper level, which assists in the meaning^(17)^.

The evidence collected in this regard suggests that a task that favors a deeper level of processing and that at the same time demands a greater cognitive effort, allows OAs to use their limited cognitive resources more effectively, a situation that would enable them to initiate a relevant and elaborate encoding process that they are not capable of achieving by themselves^(18,19)^.

Fu et al.^(13)^ developed a task to experimentally control the conditions in which the encoding of information in memory occurred, varying the depth with which the participants processed a series of words and the proportion of cognitive effort committed to the encoding decision; all this aims at examining the effect of both factors on information recall. Although there are two versions of the task, one in English and the other in Dutch, up to now no task proposal has been designed in Spanish.

Given the relevance of psycholinguistic and cultural adaptations for Spanish speakers, increasingly numerous populations globally, it will be pertinent to have a task in the language and culture where it will subsequently be administered^(20,21)^. For this reason, this study was designed to develop and obtain evidence of validity for an experimental task of verbal EM in Spanish to provide a useful tool, aligned with the OA perspective, in the search for measures that allow compensating the deterioration of the EM product of cognitive aging.

## METHOD

This research is part of a project duly approved by the Research Ethics Committee of Universidade Federal de Santa Maria (UFSM), Brazil, with registration number 3.006.101.

The proposed task in Spanish considered the ES perspective as a theoretical reference^(12)^, while for the general structure, we considered the factors and design used in the study by Fu et al.^(13)^. Therefore, its administration supports a 2 x 3 intra-subject factorial design, in which the *level of processing* (deep vs superficial) is manipulated combined with three degrees of *cognitive effort* (low vs medium vs high) during the incidental encoding of words. The procedures followed for its construction are presented below.

### Construction of the processing blocks

The experimental task was organized to detect differences in the ability to encode words in episodic memory, according to the type of information to be processed (semantic versus perceptual) and the degree of relative effort involved in its encoding (low, medium, and high). A block structure was used in its elaboration, proceeding as follows:

#### Deep encoding

Deep encoding involves the pairing between a target to encode and two response options, a semantically associated word, or a distractor.

**Figure 1:**
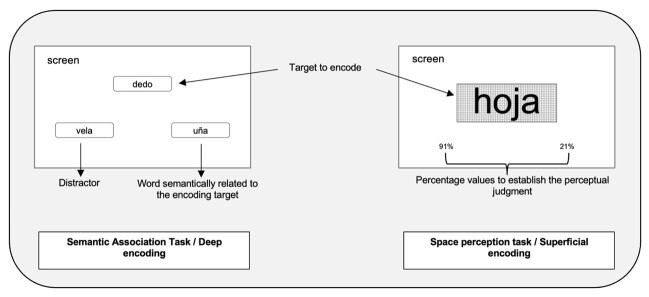
Example of semantic association task (deep encoding) and space perception task (superficial encoding)

The stimuli are presented simultaneously on a screen, always maintaining the same spatial location (target at the top; associated word and distractor at the bottom), as shown in Figure 1. It is instructed to indicate, by pressing a key, the word semantically associated with the target. To avoid response bias, the position of the associated word and the distractor was randomized.

Three deep processing blocks were designed, each with a different demand for encoding effort. The blocks were built progressively. The details related to the selection of the words as well as the variation of the semantic cognitive effort are presented below.

##### Criteria for the selection of deep encoding targets, associated words, and distractors

For the selection of the targets, the concrete “nouns” category was considered so that they were highly imaginable and easy to recognize. The following psycholinguistic properties were also considered, i) the Lexical Availability Index (LAI), and ii) the metrics of the words. The LAI allows quantifying the ease and speed to evoke the different lexical units in a given situation^(22)^.

Thus, **block one** included targets that i) were within the first 100 words with the highest LAI values according to the Dictionary of Lexical Availability in Chilean Students^(22)^, and ii) had a metric equal to or less than three syllables. All words belonging to more than one grammatical category and/or that were considered hypernyms were discarded.

**Block two** included two subgroups of equivalent targets, with 50% of stimuli meeting the block one criteria and the remaining percentage meeting the block three criteria.

Finally, in **block three**, it was decided to choose another group of targets that had to present the lowest LAI values and have a length greater than three syllables.

In the case of associated words, these were selected after administering a discrete word-free association task^(23)^ to a sample of 30 people selected for convenience, of different gender (Female = 73.3%; Male = 26.7%); age (M= 54 years, min. 45 and max. 65) and level of education (M= 11.2 years, min. 8 and max. 19). Each participant was given a paper sheet with the targets selected in the previous step. They were instructed to evoke the first word that came to mind before each target. There was a requirement established that the answer be a single word or concept.

With the cohort of alternatives that the participants activated for each of the targets, frequency tables were made. It should be noted that their choice was made taking care that they contributed to establishing differences in the strength of association between each semantic block.

For the targets in **block one**, the associated word that appeared with the highest frequency in the distribution was selected. For those included in **block two**, the word with an intermediate frequency of occurrence was selected, while for the targets in **block three**, the word with the lowest frequency was selected.

To provide greater consistency to the differentiation of the strength of association, a sample of 10 participants of both genders was recruited for convenience, ≥ to 55 years old (M= 64.1; SD= 4.6), with a level of education ≥ to 4 years of formal education (M= 9.1; SD= 2.6), with no history of neurological or psychiatric-based pathology or disease, who were instructed to quantify how related the target was to their corresponding associated word, using a Likert scale ranging from one (no association) to seven (very associated).

After selecting the targets with their respective associated words, a third word was included that worked as a distractor. This had to be part of the 100 words with the highest LAI not considered in the previous blocks and present a metric like the encoding target.

##### Criteria for varying the demand for semantic cognitive effort in the deep encoding blocks

Three variables were considered i) lexical availability; ii metrics of the encoding targets, and iii) strength of semantic association between the words. The basic premise is that the more available a word is, the less metric it has and the more associated it is with another, likely, the demand for cognitive effort to make the encoding decision will also be less, and vice versa.

Consequently, the graduation in the effort demand was established as **block one** demands a low cognitive effort, while **blocks two** and **three** demand a medium and a high effort, respectively.

### Superficial encoding

Superficial encoding involves establishing a judgment based on the perception of the space occupied by a written word (target to encode) within a grid, considering two response options, a higher percentage value and a lower magnitude.

The stimuli are presented simultaneously on a screen, always maintaining the same spatial location (target in the central part of the grid; high and low percentage value in the lower one), as shown in Figure 1. It is instructed to estimate the percentage value which corresponds to the space that the word occupies within the grid. To avoid bias, the position of the percentage values was randomized.

Subsequently, another three blocks of work were designed, each with a different demand of effort for encoding. The details related to the selection of the targets and the variation of the perceptual cognitive effort are presented below.

Criteria for the selection of superficial encoding targets

The targets were selected after consulting the lexical availability dictionary. The criteria addressed were: i) to be a specific noun, ii) to have a metric between two and three syllables and iii) to have a high value of lexical availability. The same exclusion criteria previously presented operated in this case.

#### Variation in the demand for perceptual-cognitive effort in the superficial encoding blocks

The variation depended on the difference between the percentage values that constituted the response options. The basic premise is that the greater the magnitude of the difference, the less cognitive effort required to make the encoding decision is likely to turn out to be less, and vice versa.

To ensure that people read all the targets when performing the perceptual judgment, we decided to add 12 pseudowords to the total list of trials^(24)^. They were equally distributed among the three superficial blocks, each one made up of four pseudowords. After explaining what they consist of, the participants were instructed to count how many of them they were able to identify.

In the administration of the encoding blocks, we should highlight that a maximum of five seconds is granted between each item to provide the answer. A time of 15 seconds is also granted before starting the next block. The time considered for the transition between the deep and superficial blocks is 30 seconds.

After structuring the entire experimental task and obtaining evidence of its content validity, four specialist judges, all speech and language pathologists with training and experience of at least five years in cognitive assessment, were invited to analyze all the information regarding its structure.

Based on this input and using a four-point Likert-type scale (0= completely disagree; 1= disagree; 2= agree; 3= completely agree), they completed a questionnaire in which they expressed their agreement on the combined manipulation of both factors and the precision of the instructions. All the judges were duly informed about the purpose of their participation and voluntarily signed the Informed Consent Term.

After accepting the recommendations of the judges, the test was checked in a pilot application on a sample of six cognitively normal OAs, female, between 62 and 80 years old (M= 70.5 years; SD= 6.7) and an education level between 5 and 12 years (M= 9.17 years; SD= 3.2), who voluntarily signed the Informed Consent Term. Each participant visited the Community Center for the Elderly People. In that place, they were in front of a screen and immediately completed all the processing blocks. After concluding the tests, through a question method (probing) and paraphrasing (paraphrasing), the participants expressed in their words the perceived meaning of the instructions in each block. Subsequently, they completed a brief questionnaire referring to whether the instructions of the tasks proposed in the superficial and deep encoding blocks were (1) clear and understandable, (2) difficult to understand, (3) incomprehensible; if the examples provided, (1) facilitated the understanding of the instruction, (2) were difficult to understand, (3) incomprehensible, and if the general workflow in the experimental blocks was (1) easy to understand and perform, ( 2) difficult to understand and perform, (3) incomprehensible. At the same time, the time (in minutes) spent processing each block was recorded.

The experiment was designed in Microsoft PowerPoint from Microsoft Office 365^®^ package for Mac OS.

### Data analysis

To characterize the lexical availability and the metrics of the targets included in the deep blocks, the means and standard deviations are reported. Next, to establish whether the LAIs and the length of the targets presented statistically significant differences, an analysis of variance was performed using the one-way ANOVA test and a post hoc analysis with Bonferroni adjustment.

To obtain evidence of validity related to the content, in the first instance, the degree of agreement between the judges was analyzed regarding whether they considered that the tasks included in the experimental blocks allowed for the combined manipulation of the factors of level of processing and cognitive effort; and also if the instructions were precise to understand the instruction of each activity, all this, using Kendall's W coefficient.

Using the same coefficient, the agreement between the responses emitted by the participants after the pilot application was also analyzed in the comprehensibility of the instructions, the usefulness of the examples, and the global work dynamics of the experimental task.

Finally, to establish whether there were significant differences in the time taken to complete each experimental block, an intra-group analysis of variance was performed using the ANOVA test of one factor for repeated measures.

All the data analysis was carried out with the SPSS statistical package in its version 22 for Mac OS.

## RESULTS

### Structuring of the processing blocks

#### Deep encoding blocks

**Figure 2:**
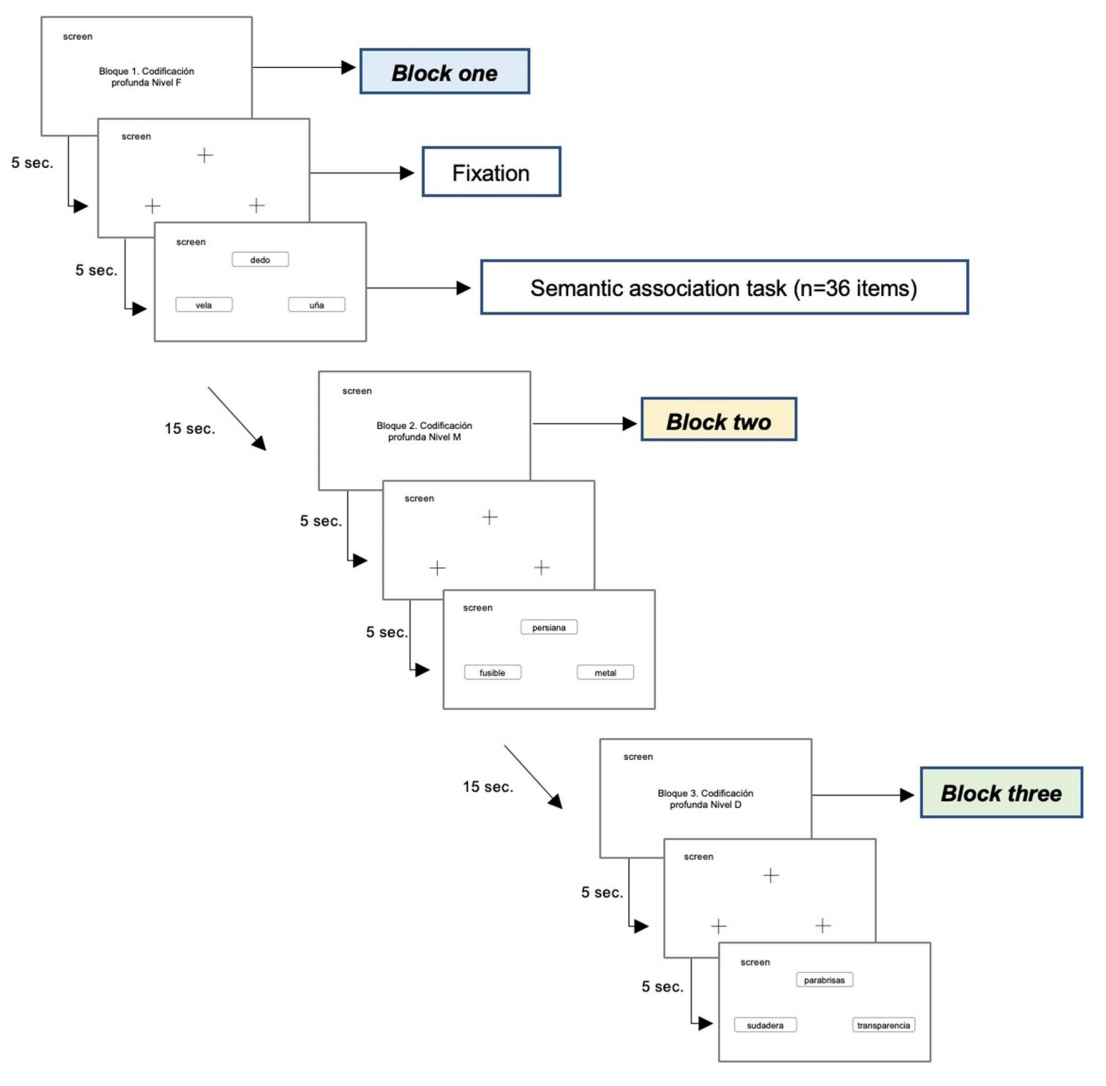
General scheme of application of the sequence of deep encoding blocks

The deep blocks included a total of 108 encoding targets, distributed in three blocks (see Figure 2).

We present below, the results of the selection of targets that were part of each of the deep blocks after meeting the respective criteria and the result obtained in the variation of cognitive effort:

##### Selection of targets for deep encoding

Based on the defined criteria, a total of 53 specific nouns were selected for **block one**. Seven of them were discarded because they belonged to more than one grammatical category, five because they were repeated, and five because they had more than three syllables, leaving a total of 36 targets for encoding with high values of LAI (M= 0.33) and short meter (M= 2.14 syllables).

**Block two** incorporated another 36 targets to code. The first subgroup included 18 concrete nouns with high LAI values (M= 0.16) and short lengths (M= 2.5 syllables). The remaining 18 presented a lower LAI (M= 0.01) and a higher metric (M= 4.06 syllables).

In contrast to the targets included in the preceding blocks, those included in **block three** present higher metrics (M= 3.86 syllables) and lower LAI values (M= 0.001).

Table 1 shows that after performing a comparative analysis using the one-way ANOVA test, it was possible to verify the existence of a statistically significant difference between the **lexical availability** of the targets that were part of the three deep blocks.

**Table 1:** Comparison of the lexical availability and metrics of the targets in the deep encoding blocks

Psycholinguistic Properties	Deep Encoding Blocks			
	B1	B2	B3	*ANOVA* *
LAI				
Mean (SD)	0.33 (0.21)	0.08 (0.12)	0.001 (0.002)	F= 52.89
				p< 0.001
Metric a				
Mean (SD)	2.14 (0.54)	3.28 (0.94)	3.86 (0.68)	F= 50.14
				p< 0.001

* one-way ANOVA test;

a measure as number of syllables

**Caption:** LAI= Lexical Availability Index; SD= Standard deviation; B= block

The post hoc analysis, adjusted with Bonferroni, shows that the LAI of the targets included in **block one** differs significantly from the LAI of the targets in **blocks two** and **three** (p≤ 0.001, in both cases). When comparing the availability between **blocks two** and **three**, there are also statistically significant differences (p= 0.038).

Regarding the metrics, the comparative analysis revealed the existence of a statistically significant difference in the length of the words included in the three deep blocks. The post hoc analysis also showed that the length of the targets in **block one** differed significantly from the length of the targets included in **blocks two** and **three** (p≤ 0.001 in both cases). The same trend is observed when comparing the length of the targets of **block two** with those of **block three** (p= 0.004) (see Table 1).

Variation in the demand for cognitive effort in the deep encoding blocks

As mentioned, there was a significant difference in the LAI and the **metrics** of the targets included in each deep block. Regarding the consistency in the differentiation of the **association strength** (see section 1.1.1), it was obtained that the average semantic association for **blocks one, two, and three** was 6.22 (0.234); 5.61 (0.338), and 4.97 (0.323), respectively. With a Fr (2) = 18.2; p≤ 0.001, it was possible to verify that there were statistically significant differences between each deep block in the association strength.

**Figure 3:**
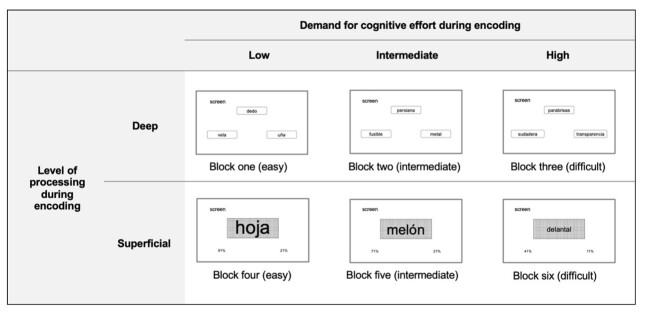
Example of a deep (semantic) and superficial (perceptive) encoding task, according to the demand for cognitive effort

Consequently, **block one**, categorized as easy, incorporated targets with high LAI, short metrics, and words strongly associated with the encoding target; on the other hand, **block two**, considered intermediate, included targets with intermediate availability, metrics, and association strength, while **block three**, classified as difficult, brought together targets with low LAI, higher metrics, and weakly associated words (see Figure 3).

### Superficial encoding blocks

**Figure 4:**
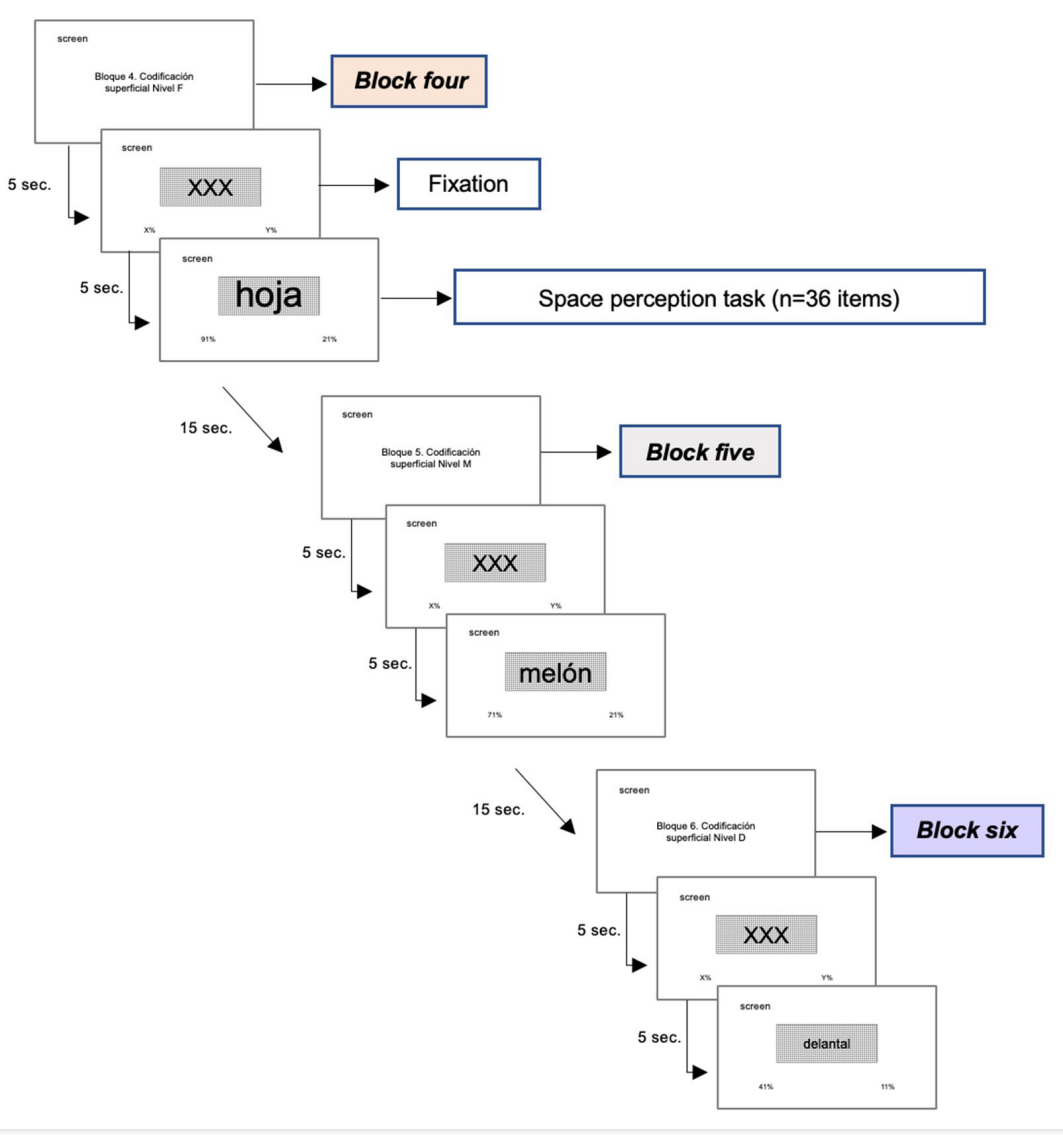
General scheme of application of the sequence of superficial encoding blocks

A total of 108 targets that met the defined criteria were selected to maintain equivalence in the total number of targets between both sets of blocks, distributed in groups of 36 among the three superficial blocks (see Figure 4).

#### Selection of targets for superficial encoding

Table 2 reports that the LAI of the nouns included in the superficial blocks did not show significant differences. However, in the metrics, it differs significantly. The post hoc analysis, in this case, revealed that **block four** differs from **blocks five** and **six** (p≤ 0.001, in both cases). However, **block five** did not show significant differences with **block six** (p= 0.564).

**Table 2:** Comparison of lexical availability and metrics of targets in superficial encoding blocks

**Psycholinguistic Properties**	**Superficial Encoding Blocks**			
	**B4**	**B5**	**B6**	***ANOVA*** *
LAI				
Mean (SD)	0.06 (0.06)	0.04 (0.03)	0.04 (0.04)	F= 1.11
				p= 0.320
Metric a				
Mean (SD)	2.00 (00)	2.69 (0.46)	2.81 (0.40)	F= 54.25
				p< 0.001

* one-way ANOVA test;

a measure as number of syllables

**Caption:** LAI= Lexical Availability Index; SD= Standard deviation; B= block

#### Variation in the demand for perceptual-cognitive effort in the superficial encoding blocks

In **block four** - classified as easy, in **block five** - considered as intermediate and in **block six** - classified as difficult, the magnitude of the difference between the percentages that represented the response options before the perceptual judgment was 70%, 50%, and 30%, respectively (see Figure 3).

The pseudowords were equally distributed in **block four**, *“baita”, “mengo”, “esmo” and “miendo”* were included; in **block five** *“sitaen”, “paesma”, “diconsias”* and *“perliteble”*; and in **block six** *“camendo”, “pacosena”, “entosame” and “deteraco”*.

### Analysis of expert judges

After completing the questionnaire, it was possible to verify a good agreement between the judges regarding i) the experimental task of verbal EM in Spanish admits the combined manipulation of the factors level of depth and cognitive effort during the incidental learning of words (W= 0.607; p= 0.046), and ii) the instructions are clear and precise to understand the dynamics of each task (W= 0.750; p= 0.029). However, within the observations, they suggested the need to reinforce with examples before beginning the administration.

### Pilot study

Regarding the results of the pilot application, a good agreement was observed between the OAs when considering that i) the instructions for the deep and superficial level of processing were clear and understandable, ii) the examples used facilitated understanding of the instructions, and iii) the work dynamics in each block was easy to understand and carry out (W= 0.514; p= 0.015).

Despite positively evaluating the comprehension of the instructions, the interviewees observed that the instruction at the superficial level was more difficult to understand. Through inquiry and paraphrasing, the participants tended to confuse what was requested. In this case, we decided to include the percentages that represented the response options as part of the slogan, as *“Here we have “X%” and “Y%”, you must choose between these two values, the one that most approximates the space occupied by the written word within the rectangle*”. We clarify that the evaluated person should not make calculations, but rather choose an alternative between the two options presented.

Accepting the recommendations of the judges and the evaluation of the piloting process, the final version of the experimental task of verbal EM in Spanish was structured.

#### Times reached after completing the deep and superficial encoding blocks

As can be seen in Table 3, the levels of complexity significantly affected the time that each participant invested in completing the task, both in the deep (F = 21.65; p < 0.001) and superficial blocks (F = 20.58; p < 0.001).

**Table 3:** Comparison of the times reached after completing each of the encoding blocks

**Parameter**	**Encoding blocks**							
	**Deep**				**Superficial**			
	**B1**	**B2**	**B3**	***ANOVA*** *	**B4**	**B5**	**B6**	ANOVA*
Time (minutes) Mean (SD)	1.65 (0.73)	2.44 (1.08)	3.53 (1.59)	F = 21.65	2.05 (0.32)	2.45 (0.48)	2.97 (0.49)	F = 20.58
				p< 0.001				p< 0.001

* = ANOVA test of one factor for repeated measures

**Caption:** B= block

## DISCUSSION

Experimental research paradigms seek to deepen the effects of age on EM performance, to demonstrate how the environmental support provided during encoding translates into positive benefits for recovery.

Considering this background, this research sought to develop and validate a verbal EM task in Spanish that combined to manipulate the environmental factors, level of processing, and cognitive effort during the encoding of words in memory.

The general structure of the proposal was inspired by the design used by Fu et al.^(13)^, whose original version is in Dutch.

As it is an activity designed for use in research that considers the participation of Spanish-speaking OAs, the construction of the tasks, the selection of the stimuli, and the variation in the degree of cognitive effort in both the deep and superficial blocks considered psycholinguistic variables adjusted to the Spanish of Chile.

In the structuring of the deep blocks, the differentiation in the LAI, in the metric, and the strength of semantic association between the words included in each block, turned out to be useful variables to produce differences in cognitive effort. It was possible to appreciate that the processing of a target demanded a low effort to make the encoding decision when (i) it presented a high LAI, (ii) it was of short metric and (iii) it was strongly associated with the word that the participants had to choose after semantic judgment. This situation was consistent with the time that the participants invested in completing each deep block.

Along the same lines, previous research suggests that the shorter and more available a word is and the greater its strength of association with another, the easier the lexical-semantic processing will be^(25,26)^, being possible to expect a more efficient encoding.

Thus, the difficulty posed by a task is directly related to the degree of cognitive effort expended to solve it. The tendency is that the more difficult it is, the effort required to solve it is also greater, and vice versa^(27)^.

In the case of the superficial blocks, the perception task was structured thinking that the difference between the percentages would allow establishing variations in the cognitive effort. The data confirm this, since the time invested in completing the perceptual task, as in the deep blocks, was directly proportional to the complexity and effort required.

Despite the fact that there was a statistically significant difference in the metrics of the targets that were part of the superficial blocks, it should be mentioned that this finding did not impact the utility of the perceptual task, since the IDL and the metrics were not determining variables in the selection of the encoding targets or in the differentiation of the complexity between the superficial blocks.

Another interesting aspect to discuss is related to intentionality in recall. It is recognized that the existence of an explicit intention to learn allows individuals to acquire knowledge. However, incidental learning, which until now has received less attention, is being increasingly considered in the research field with older people, because this type of learning is what usually occurs in daily life situations, where you are not always aware and there is a willingness to learn new information^(14,28)^.

The results are interesting, though, it is possible to identify a limitation related to the field of applicability of these results. Given the characteristics of any experimental situation, in which there is a strictly controlled environment, the plausibility of its use is mainly restricted to the research field.

Accordingly, this study adds to the efforts of others that have demonstrated the benefits of manipulating environmental factors during the encoding of information on its subsequent retrieval^(13,29)^. However, it is important to advance the applicability of the empirical results towards more daily activities carried out by OAs, so that activities of this type acquire greater ecological validity^(30)^. Therefore, to develop methods capable of combining quantitative rigor with clinical significance, further progress in dialogue between investigators and clinicians is required.

We must also mention that it is a proposed task in Spanish so for its proper use in future research, the necessary psycholinguistic adaptations must be made, always considering the cultural variants of each population.

Finally, the projections of this study are oriented towards evaluating the effect that controlled encoding generates in the retrieval of information from memory, considering not only the manipulation of environmental factors such as those included in this study but also evaluating how the cognitive resources that older people have may mediate this effect.

## CONCLUSION

This research shows a proposal for an experimental verbal episodic memory task in Spanish, which allows controlling the conditions in which encoding occurs, a highly significant aspect in the assessment of episodic memory, also revealing robust methodological elements that support its validity and replicability.

The construction of experimental instruments and tasks constitutes a determining factor to advance from parsimonious and strictly controlled models, towards their applicability in clinical settings. In this regard, it is essential to note that, in this case, the evaluation of specialist and non-specialist judges, as well as the results obtained after its application, provided evidence of content validity to the proposed task, reaching a first milestone that it should move towards a greater contribution to routine practice.

Consequently, the factorial combination of the processing level and the cognitive effort during encoding make this task a viable tool to implement in research that reveals the significance of environmental factors as facilitating or compensating aspects for the differences associated with age in the performance of the EM.
