# Supplementary material for: Development and validation of an experimental verbal Episodic Memory task in Spanish
Source: Codas. 2023 Sep 18;35(4):e20220067. doi: 10.1590/2317-1782/20232022067en (PMC10546922; doi:10.1590/2317-1782/20232022067en)
Supplement: Translation

# Translation: Desarrollo y validación de una tarea experimental de Memoria Episódica verbal en español

**DOI:** 10.1590/2317-1782/20232022067es

**Authors:** Gabriel Urrutia Urrutia, Pedro García Montenegro, Karina Carlesso Pagliarin, Márcia Keske-Soares

**Affiliations:** 1 Departamento de Ciencias de la Fonoaudiología, Facultad de Ciencias de la Salud, Universidad de Talca - UTALCA - Talca (VII Región del Maule), Chile.; 2 Programa de Pós-graduação em Distúrbios da Comunicação Humana, Universidade Federal de Santa Maria - UFSM - Santa Maria (RS), Brasil.

**Keywords:** es: Almacenamiento y Recuperación de la Información, Consolidación de la Memoria, Memoria Episódica, Semántica, Validez de los Resultados, Envejecimiento Cognitivo

## Abstract

**Propósito:**

Elaborar y validar una tarea experimental de memoria episódica verbal en español.

**Método:**

Se elaboraron seis bloques de codificación: tres profundos y tres superficiales, cado uno con distintas demandas de esfuerzo cognitivo. Los bloques fueron revisados por cuatro jueces expertos y examinados en una aplicación piloto. Se evaluó la concordancia respecto a si la tarea permitía manipular combinadamente el nivel de procesamiento y el esfuerzo cognitivo durante la codificación incidental de palabras, así como la claridad de las instrucciones, ejemplos y dinámica de trabajo.

**Resultados:**

Variables como la disponibilidad léxica, metría y fuerza de asociación fueron útiles para diferenciar el esfuerzo cognitivo entre cada bloque. Los jueces concordaron que los bloques de procesamiento admiten una manipulación combinada del nivel de procesamiento y esfuerzo cognitivo y que las instrucciones son precisas. Luego del pilotaje, los participantes concordaron que las instrucciones, ejemplos y forma de trabajo eran fácil de comprender y realizar.

**Conclusión:**

Los resultados proporcionan evidencia de validez relacionada con el contenido para la tarea experimental propuesta, transformándose con ello en una alternativa viable de considerar en investigaciones orientadas a identificar factores ambientales que contribuyan a compensar los defectos que muestra la memoria episódica con la edad.

## INTRODUCCIÓN

La memoria episódica (ME) es responsable de codificar, almacenar y recuperar conscientemente eventos pasados experimentados personalmente^(1)^. Es uno de los primeros dominios que muestra un declive desde el inicio del envejecimiento^(2)^, vinculado con un ineficiente funcionamiento de los procesos de codificación y recuperación^(3,4)^.

La *codificación* es el proceso de la memoria que participa en la adquisición y transformación de la información nueva que se procesa en una representación mental^(5)^. Este proceso suele demandar muchos recursos de procesamiento, como la capacidad selectiva de la atención, la velocidad de procesamiento y el control ejecutivo de la memoria de trabajo^(6)^. Lamentablemente, con la edad dichos procesos suelen mostrar un declive en su funcionamiento^(7-9)^.

Conforme a la hipótesis de recursos limitados^(10)^, los Adultos Mayores (AM) exhibirían una menor capacidad para iniciar espontáneamente un procesamiento cognitivo relevante al momento de codificar nueva información en la memoria.

Según la teoría de cognición distribuida, el rendimiento cognitivo sería el resultado de una interacción entre componentes internos (cognitivos) y externos (ambientales)^(11)^. Acorde con dicho planteamiento, el procesamiento de información comenzaría "de arriba hacia abajo” movilizado por las intenciones actuales de las personas, pero en parte también por la estimulación externa que impulsa un procesamiento desde “abajo hacia arriba”. Al unir estas ideas, es plausible hipotetizar que algunas deficiencias relacionadas con la edad en el procesamiento autoiniciado (de arriba hacia abajo) podrían reducirse al aumentar el componente ascendente impulsado desde el exterior en la forma de un Apoyo Ambiental (AA)^(12)^.

La evidencia aportada por estudios conductuales ha demostrado que proporcionar AA durante la codificación produce efectos positivos en el recuerdo^(13-15)^. El AA se entiende entonces como un soporte externo de rendimiento que actúa manipulando las demandas de una tarea cognitiva para favorecer un procesamiento más eficaz de la información^(12)^.

En esta línea, se han propuesto dos factores ambientales que podrían favorecer mecanismos de compensación para la ME, a saber, el *esfuerzo cognitivo* y el *nivel de procesamiento de la información*.

El primero refiere a la proporción de procesamiento que una persona compromete ante una tarea desafiante^(16)^. El nivel de procesamiento alude al grado de profundidad con que se procesa la información, variando desde un nivel superficial, que atiende a elementos más perceptuales, hasta un nivel más profundo, que atiende al significado^(17)^.

La evidencia recopilada al respecto sugiere que una tarea que favorece un nivel más profundo de procesamiento y que a la vez demanda un mayor esfuerzo cognitivo, permite que los AM utilicen con mayor eficacia los recursos cognitivos limitados que presentan, situación que los facultaría para iniciar un proceso de codificación relevante y elaborado que, por sí solos, no son capaces de alcanzar^(18,19)^.

Fu et al.^(13)^ desarrollaron una tarea para controlar experimentalmente las condiciones en las que ocurría la codificación de información en la memoria, variando la profundidad con que los participantes procesaban una serie de palabras y la proporción de esfuerzo cognitivo comprometido en la decisión de codificación; todo ello, con el propósito de examinar el efecto de ambos factores en el recuerdo de información. Si bien existen dos versiones de la mencionada tarea, una en inglés y otra en holandés, hasta el momento no se ha diseñado una propuesta de tarea en español.

Dado la relevancia de las adaptaciones psicolingüísticas y culturales para los hispanohablantes, una población cada vez más numerosa a nivel global, será pertinente disponer de una tarea en el propio idioma y cultura donde posteriormente se administrará^(20,21)^. Por tal razón, el presente estudio fue diseñado con el propósito de elaborar y obtener evidencia de validez para una tarea experimental de ME verbal en español, a fin de proporcionar una herramienta útil, alineada con la perspectiva del AA, en la búsqueda de medidas que permitan compensar el deterioro de la ME producto del envejecimiento cognitivo.

## MÉTODO

Esta investigación forma parte de un proyecto debidamente aprobado por el Comité de Ética en Investigación de la Universidade Federal de Santa Maria (UFSM), Brasil, con el número de registro 3.006.101.

La tarea en español propuesta consideró como referente teórico la perspectiva de AA^(12)^, mientras que para la estructura general se tomó en cuenta los factores y el diseño utilizado en el estudio de Fu et al.^(13)^. Por tanto, su administración soporta un diseño factorial 2 x 3 intrasujeto, donde se manipula combinadamente el *nivel de procesamiento de la información* (profundo vs superficial) con 3 grados de *esfuerzo cognitivo* durante la codificación incidental de palabras (bajo, medio y alto). A continuación, se presentan los procedimientos efectuados para su construcción.

### Construcción de los bloques de procesamiento

La tarea experimental se organizó con el objeto de detectar diferencias en la capacidad de codificar palabras en la memoria episódica, según el tipo de información a procesar (semántica versus perceptual) y el grado de esfuerzo relativo implicado en su codificación (bajo, medio y alto). En su elaboración se utilizó una estructura de bloques, procediendo de la siguiente manera:

#### Codificación profunda

La codificación profunda implica el emparejamiento entre un target para codificar y dos opciones de respuesta: una palabra semánticamente asociada o un distractor.

Los estímulos son presentados simultáneamente en una pantalla, manteniendo siempre la misma ubicación espacial (target en la parte superior; palabra asociada y distractor en la inferior), tal como se muestra en la Figura 1. Se instruye indicar, presionando una tecla, la palabra semánticamente asociada al target. Para evitar un sesgo en las respuestas, la posición de la palabra asociada y el distractor fue aleatorizada.

**Figura 1:** Ejemplo tarea de asociación semántica (codificación profunda) y tarea de percepción de espacio (codificación superficial)

Se diseñaron 3 bloques de procesamiento profundo, cada uno con una demanda diferente de esfuerzo para la codificación. Los bloques se construyeron de modo progresivo. Los detalles relativos a la selección de las palabras, así como la variación del esfuerzo cognitivo semántico son presentados a continuación.

##### Criterios para la selección de los targets de codificación profunda, de las palabras asociadas y distractores

Para la selección de los targets, se consideró la categoría “sustantivos” concretos, para que fueran altamente imaginables y fáciles de reconocer. También se consideraron las siguientes propiedades psicolingüísticas: i) el Índice de Disponibilidad Léxica (IDL), y ii) la metría de las palabras. El IDL permite cuantificar la facilidad y rapidez para evocar las distintas unidades léxicas ante una determinada situación^(22)^.

De esta manera, el ***bloque uno*** incluyó targets que: i) se encontraban dentro de las primeras 100 palabras con los valores más altos de IDL según el diccionario de Disponibilidad Léxica en Estudiantes Chilenos^(22)^, y ii) poseían una metría igual o menor a tres sílabas. Se descartaron todas las palabras pertenecientes a más de una categoría gramatical y/o que fueran consideradas como hiperónimos.

El ***bloque dos*** incluyó dos subgrupos de targets equivalentes, con un 50% de estímulos que cumplían con los criterios del bloque uno y el porcentaje restante con los criterios del bloque tres.

Finalmente, en el ***bloque tres,*** se optó por elegir otro grupo de targets que debían: i) presentar los valores de IDL más bajos y poseer una longitud superior a tres sílabas.

En el caso de las palabras asociadas, estas fueron seleccionadas después de administrar una tarea de libre asociación discreta de palabras^(23)^, a una muestra de 30 personas seleccionadas por conveniencia, de diferente sexo (Femenino= 73,3%; Masculino= 26,7%); edad (M= 54 años, mín. 45 y máx. 65) y nivel de escolaridad (M= 11,2 años, mín. 8 y máx. 19). A cada participante se proporcionó una hoja con los targets seleccionados en el paso anterior. Se instruyó evocar la primera palabra que les viniera a la mente ante cada target. Se estableció como requisito, que la respuesta fuera una sola palabra o concepto.

Con la cohorte de alternativas que los participantes activaron para cada uno de los targets, se confeccionaron tablas de frecuencia. Cabe destacar que su elección se realizó cuidando que contribuyero n a establecer diferencias en la fuerza de asociación entre cada bloque semántico.

Para los targets del **bloque uno**, se seleccionó la palabra asociada que en la distribución apareció con la frecuencia más alta. Para los incluidos en el **bloque dos**, se seleccionó aquella que presentaba una frecuencia intermedia de aparición, mientras que para los targets del **bloque tres** se seleccionó la palabra que presentaba la frecuencia más baja.

Para proporcionar mayor consistencia a la diferenciación de la fuerza de asociación se reclutó, por conveniencia, una muestra de 10 participantes de ambos sexos, con una edad ≥ a 55 años (M= 64,1; DE= 4,6), con un nivel de educación ≥ a 4 años de instrucción formal (M= 9,1; DE= 2,6), sin antecedentes de patología o enfermedad de base neurológica o psiquiátrica, quienes fueron instruidos en cuantificar qué tan relacionado se encontraba el target con su correspondiente palabra asociada, utilizando una escala Likert que oscilaba entre uno (sin asociación) y siete (muy asociadas).

Luego de seleccionar los targets con sus respectivas palabras asociadas, se incluyó una tercera palabra que funcionaba como distractor. Este debía formar parte de las 100 palabras con IDL más alto no consideradas en los bloques anteriores y presentar una metría similar al target de codificación.

##### Criterios para variar la demanda de esfuerzo cognitivo semántico en los bloques de codificación profunda

Se consideraron tres variables: i) *disponibilidad léxica*; ii *metría* de los targets de codificación, y iii) fuerza *de asociación semántica* entre las palabras. La premisa básica es que mientras más disponible se encuentre una palabra; mientras menor metría posea y mientras más asociada se encuentre con otra, es probable que la demanda de esfuerzo cognitivo para tomar la decisión de codificación sea menor también, y viceversa.

En consecuencia, la graduación en la demanda de esfuerzo quedó establecida de la siguiente manera: el ***bloque uno*** demanda un bajo esfuerzo cognitivo, mientras que el ***bloque dos y tres*** demandan un mediano y un alto esfuerzo, respectivamente.

### Codificación superficial

La codificación superficial implica establecer un juicio basado en la percepción del espacio que ocupa una palabra escrita (target para codificar) dentro de una cuadrícula, considerando dos opciones de respuesta: un valor porcentual de mayor y otro de menor magnitud.

Los estímulos son presentados simultáneamente en una pantalla, manteniendo siempre la misma ubicación espacial (target en la parte central de la cuadrícula; valor porcentual alto y bajo en la inferior), tal como se muestra en la Figura 1. Se instruye estimar el valor porcentual que corresponde al espacio que ocupa la palabra dentro de la cuadrícula. Para evitar sesgos, la posición de los valores porcentuales fue aleatorizada.

Consecuentemente, se diseñaron otros 3 bloques de trabajo, cada uno con una demanda diferente de esfuerzo para la codificación. Los detalles relativos a la selección de los targets, así como la variación del esfuerzo cognitivo perceptual son presentados a continuación.

#### Criterios para la selección de los targets de codificación superficial

Los targets fueron seleccionados después de consultar el diccionario de disponibilidad léxica anteriormente citado. Los criterios atendidos fueron: i) ser un sustantivo concreto, ii) poseer una metría entre 2 y 3 sílabas y iii) poseer un valor de disponibilidad léxica alto. Los mismos criterios de exclusión anteriormente presentados operaron en este caso.

#### Variación en la demanda de esfuerzo cognitivo perceptual en los bloques de codificación superficial

La variación dependió de la diferencia entre los valores porcentuales que constituían las opciones de respuesta. La premisa básica es que mientras mayor es la magnitud de la diferencia, probablemente el esfuerzo cognitivo que se requiere para tomar la decisión de codificación resulte ser menor, y viceversa.

Para garantizar que las personas lean todos los targets al realizar el juicio perceptual, se optó por adicionar 12 pseudopalabras a la lista total de ensayos^(24)^. Estas se distribuyeron equitativamente entre los tres bloques superficiales, quedando cada uno conformado por cuatro pseudopalabras. Después de explicar en qué consisten, se instruyó a los participantes en contar cuántas de ellas eran capaces de identificar.

En relación con la administración de los bloques de codificación, cabe destacar que se concede un máximo de 5 segundos entre cada ítem para proporcionar la respuesta. Se otorga también un tiempo de 15 segundos antes de iniciar el siguiente bloque. El tiempo considerado para la transición entre los bloques profundos y los superficiales es de 30 segundos.

Luego de estructurar la tarea experimental completa y con el fin de obtener evidencia de su validez de contenido, se invitó a cuatro jueces especialistas, todos fonoaudiólogos con formación y experiencia de al menos cinco años en evaluación cognitiva, para que analizaran toda la información referente a su estructura.

A partir de dicho insumo, y utilizando un escalamiento tipo Likert de cuatro puntos (0= completamente en desacuerdo; 1= en desacuerdo; 2= de acuerdo; 3= completamente de acuerdo), completaron un cuestionario en el que expresaron su grado de acuerdo sobre la manipulación combinada de ambos factores y la precisión de las instrucciones. Todos los jueces fueron debidamente ilustrados sobre el propósito de su participación y firmaron voluntariamente el Término de Consentimiento Libre e Informado.

Luego de acoger las recomendaciones de los jueces, la prueba fue testeada en una aplicación piloto a una muestra de seis AM cognitivamente normales, de sexo femenino, con edades entre 62 y 80 años (M= 70,5 años; DE= 6,7) y una escolaridad entre 5 y 12 años (M= 9,17 años; DE= 3,2), quienes voluntariamente firmaron el Término de Consentimiento Libre e Informado. Cada participante fue visitado en el centro Comunitario de Adulto Mayor al que asistía. En dicho lugar fue dispuesto frente a una pantalla y enseguida completaron todos los bloques de procesamiento. Luego de concluir los ensayos, mediante un método de pregunta (*probing*) y parafraseo (*paraphrasing*), los participantes expresaron con sus propias palabras el significado percibido en las instrucciones de cada bloque. Posteriormente, completaron un breve cuestionario refiriéndose a si las instrucciones de las tareas propuestas en los bloques de codificación superficial y profunda eran: (1) clara y comprensible, (2) difícil de entender, (3) incomprensible; si los *ejemplos* proporcionados: (1) facilitan la comprensión de la instrucción, (2) eran difíciles de entender, (3) incomprensibles, y si la *dinámica de trabajo* general en los bloques experimentales era (1) fácil de comprender y realizar, (2) difícil de comprender y realizar, (3) incomprensible. A la vez, se registró el tiempo (en minutos) empleado en el procesamiento de cada bloque.

El experimento fue diseñado en Microsoft PowerPoint del paquete Microsoft Office 365^®^ para OS Mac.

### Análisis de datos

Para caracterizar la disponibilidad léxica y la metría de los targets incluidos en los bloques profundos, se reportan las medias y desviaciones estándar. Enseguida, para establecer si los IDLs y la longitud de dichos targets presentaban diferencias estadísticamente significativas, se realizó un análisis de varianza por medio de la prueba ANOVA de una vía y un análisis post hoc con ajuste de Bonferroni.

Para obtener evidencia de validez relacionada con el contenido, en una primera instancia se analizó el grado de acuerdo entre los jueces respecto a si consideraban que las tareas incluidas en los bloques experimentales admitían la manipulación combinada de los factores nivel de procesamiento y esfuerzo cognitivo; y también si las instrucciones eran precisas para comprender la consigna de cada actividad, todo ello, por medio del coeficiente W de Kendall.

Utilizando el mismo coeficiente, también se analizó la concordancia entre las respuestas emitidas por los participantes luego de la aplicación piloto, en relación con la comprensibilidad de las instrucciones, la utilidad de los ejemplos y la dinámica de trabajo global de la tarea experimental.

Finalmente, para establecer si existían diferencias significativas en el tiempo empleado para completar cada bloque experimental, se realizó un análisis de varianza intra-grupal por medio de la prueba ANOVA de un factor para medidas repetidas.

Todos los análisis se realizaron con el paquete estadístico SPSS en su versión 22 para OS Mac.

## RESULTADOS

### Estructuración de los bloques de procesamiento

#### Bloques de codificación profunda

Los bloques profundos incluyeron un total de 108 *targets* de codificación, distribuidos en 3 bloques (ver Figura 2).

**Figura 2:** Esquema general de aplicación de la secuencia de bloques de codificación profunda.

A continuación, se presentan los resultados que arrojó la selección de los targets que formaron parte de cada uno de los bloques profundos después de atender los respectivos criterios, así como el resultado obtenido en la variación del esfuerzo cognitivo:

##### Selección de los targets para la codificación profunda

Atendiendo a los criterios definidos, para el **bloque uno** se seleccionaron un total de 53 sustantivos concretos. De estos, se descartaron siete por pertenecer a más de una categoría gramatical, cinco por estar repetidos y cinco por presentar más de tres sílabas, quedando un total de 36 targets para codificar con valores altos de IDL (M= 0,33) y de corta metría (M= 2,14 sílabas).

El **bloque dos** incorporó otros 36 *targets* para codificar. El primer subgrupo incluyó 18 sustantivos concretos con valores altos de IDL (M= 0,16) y longitud corta (M= 2,5 sílabas). Los 18 restantes presentaron un IDL más bajo (M= 0,01) y una metría mayor (M= 4,06 sílabas).

En contraste con los *targets* incluidos en los bloques precedentes, los incluidos en el **bloque tres** presentan una mayor metría (M= 3,86 sílabas) y valores más bajos de IDL (M= 0,001).

La Tabla 1 muestra que luego de realizar un análisis comparativo mediante la prueba ANOVA de una vía, fue posible constatar la existencia de una diferencia estadísticamente significativa entre la **disponibilidad léxica** de los targets que formaron parte de los tres bloques profundos.

**Tabla 1:** Comparación de la disponibilidad léxica y metría de los targets en los bloques de codificación profunda

Propiedades Psicolingüísticas	Bloques de Codificación Profunda			
	**B1**	**B2**	**B3**	***ANOVA*** *
IDL				F= 52,89
Media (DE)	0,33 (0,21)	0,08 (0,12)	0,001 (0,002)	p< 0,001
Metría a				F= 50,14
Media (DE)	2,14 (0,54)	3,28 (0,94)	3,86 (0,68)	p< 0,001

* prueba ANOVA de una vía

a medida como número de sílabas

**Subtítulo:** IDL= Índice de Disponibilidad Léxica; DE= Desviación estándar; B= bloque

El análisis post hoc, ajustado con Bonferroni, muestra que el IDL de los targets incluidos en el **bloque uno** se diferencia significativamente del IDL de los targets del **bloque dos** y **tres** (p≤ 0,001, en ambos casos). Al comparar la disponibilidad entre el **bloque dos** y **tres**, se aprecia que también existen diferencias estadísticamente significativas (p= 0,038).

En relación con la **metría**, el análisis comparativo reveló la existencia de una diferencia estadísticamente significativa en la longitud de las palabras incluidas en los tres bloques profundos. Adicionalmente, el análisis post hoc mostró que la longitud de los targets del **bloque uno** difería significativamente de la longitud de los targets incluidos en el **bloque dos** y **tres** (p≤ 0,001 en ambos casos). La misma tendencia se observa al comparar la longitud de los targets del **bloque dos** con los del **bloque tres** (p= 0,004) (ver Tabla 1).

##### Variación en la demanda de esfuerzo cognitivo en los bloques de codificación profunda

Como se mencionó, existió una diferencia significativa en el **IDL** y en la **metría** de los targets incluidos en cada bloque profundo. En lo referente a la consistencia en la diferenciación de la **fuerza de asociación** (ver apartado 1.1.1), se obtuvo que la media de asociación semántica para los ***bloques uno, dos y tres*** fueron 6,22 (0,234); 5,61 (0,338) y 4,97 (0,323), respectivamente. Con un Fr (2) = 18,2; p≤ 0,001 fue posible constatar que hubo diferencias estadísticamente significativas entre cada bloque profundo en la fuerza de asociación.

En consecuencia, el **bloque uno**, categorizado como fácil, incorporó targets con IDL altos, de corta metría y palabras asociadas fuertemente con el target de codificación; en cambio el **bloque dos**, considerado como intermedio; incluyó targets con una disponibilidad, metría y fuerza de asociación intermedia, mientras que el **bloque tres**, catalogado como difícil, reunió targets con bajo IDL, con una metría mayor y palabras asociadas débilmente (ver Figura 3).

**Figura 3:** Ejemplo de una tarea de codificación profunda (semántica) y superficial (perceptiva), según demanda de esfuerzo cognitivo

### Bloques de codificación superficial

Con el fin de mantener una equivalencia en el número total de targets entre ambos conjuntos de bloques, se seleccionaron un total de 108 targets que cumplían con los criterios definidos, los que fueron distribuidos en grupos de 36 entre los tres bloques superficiales (ver Figura 4).

**Figura 4:** Esquema general de aplicación de la secuencia de bloques de codificación superficial

#### Selección de los targets para la codificación superficial

La Tabla 2 informa que el IDL de los sustantivos incluidos en los bloques superficiales no mostró diferencias significativas. No obstante, en relación con la **metría** se aprecia que esta difiere significativamente. El análisis post hoc, en este caso, reveló que el **bloque cuatro** se diferencia de los **bloques cinco** y **seis** (p≤ 0,001, en ambos casos). Sin embargo, el **bloque cinco** no mostró diferencias significativas con el **bloque seis** (p= 0,564).

**Tabla 2:** Comparación de la disponibilidad léxica y metría de los targets en los bloques de codificación superficial

Propiedades Psicolingüísticas	Bloques de Codificación Superficial			
	**B4**	**B5**	**B6**	***ANOVA*** *
IDL				F= 1,11
Media (DE)	0,06 (0,06)	0,04 (0,03)	0,04 (0,04)	p= 0,320
Metría a				F= 54,25
Media (DE)	2,00 (00)	2,69 (0,46)	2,81 (0,40)	p< 0,001

* prueba ANOVA de una vía;

a medida como número de sílabas

**Subtítulo:** IDL= Índice de Disponibilidad Léxica; DE= Desviación estándar; B= bloque

#### Variación en la demanda de esfuerzo cognitivo perceptual en los bloques de codificación superficial

En el **bloque cuatro** - clasificado como fácil, en el **bloque cinco** - considerado como intermedio y en el **bloque seis** - calificado como difícil, la magnitud de la diferencia entre los porcentajes que representaban las opciones de respuesta ante el juicio perceptual fue de 70%, 50% y 30%, respectivamente (ver Figura 3).

Las pseudopalabras se distribuyeron equitativamente de la siguiente manera: en el **bloque cuatro** se incluyeron “baita”, “mengo”, “esmo” y “miendo”; en el **bloque cinco** “sitaen”, “paesma”, “diconsias” y “perliteble”; y en el **bloque seis** “camendo”, “pacosena”, “entosame” y “deteraco”.

### Análisis de jueces expertos

Luego de completar el cuestionario, fue posible constatar una buena concordancia entre los jueces respecto a: i) la tarea experimental de ME verbal en español admite la manipulación combinada de los factores nivel de profundidad y esfuerzo cognitivo durante el aprendizaje incidental de palabras (W= 0,607; p= 0,046), y ii) las instrucciones son claras y precisas para comprender la dinámica de cada tarea (W= 0,750; p= 0,029). No obstante, dentro de las observaciones, sugirieron la necesidad de reforzar con ejemplos antes de comenzar la administración.

### Estudio piloto

Refiriéndose esta vez a los resultados de la aplicación piloto, se observó una buena concordancia entre los AM en el hecho de considerar que: i) las instrucciones del nivel profundo y superficial de procesamiento eran claras y comprensibles, ii) los ejemplos usados facilitaban la comprensión de la consigna, y iii) la dinámica de trabajo en cada bloque era fácil de comprender y realizar (W= 0,514; p= 0,015).

A pesar de valorar positivamente la comprensión de las instrucciones, los entrevistados observaron que la instrucción del nivel superficial era más difícil de entender. A través de la indagación y parafraseo, se apreció que los participantes tendían a confundir lo que se les solicitaba. En tal caso se optó por incluir los porcentajes que representaban las opciones de respuesta como parte de la consigna, de la siguiente manera: “*acá tenemos “X%” e “Y%”, usted debe escoger entre esos dos valores el que más se aproxima al espacio que ocupa la palabra escrita dentro del rectángulo”.* Se aclara que el evaluado no debe hacer cálculos, sino escoger una alternativa entre las dos opciones presentadas.

Acogiendo las recomendaciones de los jueces y la evaluación del proceso de pilotaje, se estructuró la versión definitiva de la tarea experimental de ME verbal en español.

#### Tiempos alcanzados luego de completar los bloques de codificación profunda y superficial

Tal como se aprecia en la Tabla 3, los niveles de complejidad afectaron significativamente el tiempo que cada participante invirtió en completar la tarea, tanto en los bloques profundos (F = 21,65; p < 0,001) como en los superficiales (F = 20,58; p < 0,001).

**Tabla 3:** Comparación de los tiempos alcanzados luego de completar cada uno de los bloques de codificación

**Parámetro**	**Bloques de codificación**							
	**Profunda**				**Superficial**			
	**B1**	**B2**	**B3**	***ANOVA*** *	**B4**	**B5**	**B6**	ANOVA*
Tiempo (minutos) Media (DE)	1,65 (0,73)	2,44 (1,08)	3,53 (1,59)	F = 21,65	2,05 (0,32)	2,45 (0,48)	2,97 (0,49)	F = 20,58
				p< 0,001				p< 0,001

* = prueba ANOVA de un factor para medidas repetidas

**Subtítulo:** B= bloque

## DISCUSIÓN

Los paradigmas experimentales de investigación buscan profundizar en los efectos que produce la edad en el rendimiento de la ME, con el propósito de demostrar cómo el apoyo ambiental proporcionados durante la codificación se traduce en beneficios positivos para la recuperación.

Considerando estos antecedentes, esta investigación persiguió elaborar y validar una tarea de ME verbal en español que manipulara combinadamente los factores ambientales nivel de procesamiento y esfuerzo cognitivo durante la codificación de palabras en la memoria.

La estructura general de la propuesta estuvo inspirada en el diseño utilizado por Fu et al^(13)^, cuya versión original es en idioma holandés.

Al tratarse de una actividad pensada para su uso en investigaciones que consideren la participación de AM hispanoparlantes, la construcción de las tareas, la selección de los estímulos y la variación en el grado de esfuerzo cognitivo tanto en los bloques profundos como en los superficiales, tomaron en cuenta variables psicolingüísticas ajustadas al idioma español de Chile.

En la estructuración de los bloques profundos, la diferenciación en el IDL, en la metría y en la fuerza de asociación semántica entre las palabras incluidas en cada bloque, resultaron ser variables útiles para producir diferencias en el esfuerzo cognitivo. Fue posible apreciar que el procesamiento de un target demandó un bajo esfuerzo para tomar la decisión de codificación cuando: (i) presentaba un IDL alto, (ii) era de corta metría y (iii) se asociaba fuertemente con la palabra que los participantes debían elegir luego del juicio semántico. Esta situación fue concordante con el tiempo que los participantes invirtieron en completar cada bloque profundo.

En esta misma línea, investigaciones previas sugieren que cuánto más corta y disponible se encuentra una palabra y mientras mayor es su fuerza de asociación con otra, más fácil será el procesamiento léxico-semántico^(25,26)^, con lo que consecuentemente sería posible esperar una codificación más eficaz.

Por consiguiente, la dificultad que plantea una tarea se relaciona directamente con el grado de esfuerzo cognitivo desplegado para resolverla. La tendencia es que mientras más difícil sea, la demanda de esfuerzo para resolverla sea también mayor, y viceversa^(27)^.

En el caso de los bloques superficiales, la tarea de percepción se estructuró pensando en que la diferencia entre los porcentajes permitiera establecer variaciones en el esfuerzo cognitivo. El tiempo invertido en completar la tarea perceptual, al igual que en los bloques profundos, fue directamente proporcional a la complejidad y a la exigencia de esfuerzo, confirmando que ambas variables fueron útiles para graduar el esfuerzo cognitivo perceptual.

A pesar de que hubo una diferencia estadísticamente significativa en la metría de los targets que formaban parte de los bloques superficiales, es preciso mencionar que dicho hallazgo no impacta en la utilidad de la tarea perceptual, pues el IDL y la metría no fueron variables determinantes en la selección de los targets de codificación, ni tampoco en la diferenciación de la complejidad entre los bloques superficiales.

Otro aspecto interesante de discutir se relaciona con la intencionalidad en el recuerdo. Se reconoce que la existencia de una intención explícita de aprender permite a los individuos adquirir conocimientos. Sin embargo, el aprendizaje incidental, que hasta ahora ha recibido menos atención, está siendo cada vez más considerado en el campo de la investigación con personas mayores, porque este tipo de aprendizaje es el que suele ocurrir en situaciones de la vida cotidiana, en las que no siempre se es consciente y existe la voluntad de aprender nueva información^(14,28)^.

A pesar de lo interesante de los hallazgos, es posible identificar una limitación relacionada con el ámbito de aplicabilidad de los resultados. Dado las características propias de cualquier situación experimental, en la que existe un ambiente estrictamente controlado, la plausibilidad de su uso se restringe fundamentalmente al ámbito investigativo.

Concordantemente, este estudio se suma a los esfuerzos de otros que han demostrado los beneficios de la manipulación de factores ambientales durante la codificación de información sobre su recuperación posterior^(13,29)^, sin embargo, es importante avanzar en la aplicabilidad de los resultados empíricos hacia actividades más cotidianas que realizan los AM, para que actividades de este tipo adquieran mayor validez ecológica^(30)^. Por consiguiente, para desarrollar métodos capaces de combinar el rigor cuantitativo con la significación clínica, se requiere progresar en un mayor diálogo entre investigadores y clínicos.

Hemos de mencionar también que se trata de una propuesta de tarea en español, por lo que, para su uso adecuado en futuras investigaciones, ha de efectuarse las necesarias las adaptaciones psicolingüísticas, considerando siempre las variantes culturales propias de cada población en particular.

Para finalizar, las proyecciones de este estudio se orientan en evaluar el efecto que la codificación controlada genera en la recuperación de información desde la memoria, considerando no solo manipulación de factores ambientales como los incluidos en este estudio, sino también evaluar cómo los recursos cognitivos que las personas mayores poseen pueden mediar dicho efecto.

## CONCLUSIÓN

Esta investigación presenta una propuesta de tarea experimental de memoria episódica verbal en español, que permite controlar las condiciones en las que ocurre la codificación, un aspecto altamente significativo en la valoración de la memoria episódica, relevando además elementos metodológicos robustos que sustentan su validez y replicabilidad.

La construcción de instrumentos y tareas experimentales constituye un factor determinante para avanzar desde modelos parsimoniosos y estrictamente controlados, hacia su aplicabilidad en entornos clínicos. En relación con ello, es fundamental rescatar que, en este caso, la valoración de los jueces especialistas y no especialistas, así como los resultados obtenidos tras su aplicación, proporcionaron evidencia de validez de contenido a la tarea propuesta, alcanzando así un primer hito que sin duda debe avanzar hacia un mayor aporte para la práctica rutinaria.

En consecuencia, la combinación factorial del nivel de procesamiento y el esfuerzo cognitivo durante la codificación, convierten a esta tarea en una herramienta viable de implementar en investigaciones que releven la significancia de los factores ambientales como aspectos facilitadores o compensatorios para las diferencias asociadas con la edad en el desempeño de la ME.
